# Bound states in the continuum in a chain of coupled Mie resonators with structural disorder: theory and experiment

**DOI:** 10.1515/nanoph-2025-0225

**Published:** 2025-09-17

**Authors:** Ravshanjon Nazarov, Denis Khanabiev, Elizaveta Chernysheva, Alexandra Dudnikova, Vyacheslav Istomin, Mikhail Sidorenko, Jinhui Shi, Ekaterina Maslova, Andrey Bogdanov, Zarina Kondratenko (Sadrieva)

**Affiliations:** Qingdao Innovation and Development Center of Harbin Engineering University, Qingdao, 266500, China; School of Physics and Engineering, 47822 ITMO University, St. Petersburg, 197101, Russia; Physics and Mathematics Lyceum 239, St. Petersburg, 191144, Russia

**Keywords:** bound states in the continuum, Mie resonances, structural disorder

## Abstract

We study the impact of structural disorder on a radiative lifetime of symmetry-protected bound state in the continuum (BIC) and a bright mode in a one-dimensional periodic chain of coupled Mie resonators. Through experimental, simulation, and theoretical approach, we reveal an unusual linear decay in the radiative quality factor of the BIC with the increase of the disorder amplitude, contrasting with the quadratic decay observed in recent studies. We also investigate modes with different symmetries and show that the behavior of the quality factor in a strongly disordered system depends on the mode’s multipolar origin. In addition, we found that in a disordered finite chain, the quality factor of Fabry–Pérot modes is governed by intraband interactions of modes. To study the behavior of localized states in disordered systems, we also analyze the emergence of Anderson localization. Our findings are pivotal for the practical application of BICs, particularly in natural and self-assembled photonic structures where structural disorder plays a crucial role.

## 1 Introduction

The concept of bound states in the continuum (BICs) was introduced in quantum mechanics in 1929 [1], and over the last decade, BICs have become a prominent phenomenon in optics [2], [3], [4], [5], [6], [7], [8], [9], [10], [11], [12], [13], [14]. BICs are nonradiative states that remain localized in the continuum of propagating waves of the environment. In theory, these states have an infinite radiative quality (*Q*) factor, giving rise to a variety of applications, including nonlinear optics [2], enhancement of nonlinear harmonic generation [3], [4], [5], optical modulators, lasing [6], [7], [8], [9], [10], sensing technologies [11], [12], and achieving strong coupling phenomena [13], [14]. However, practical aspects such as the finite size of the sample, material absorption, and defects created during the resonator fabrication limit the *Q* factor of BICs [15], [16], [17], [18]. Nanostructure fabrication techniques like ultraviolet [19] and electron-beam lithography [18] enable precise structural configurations with resolutions down to a few nanometers [20]; however, lithography often introduces surface roughness. Among various factors, scattering losses due to fabrication imperfections or disorder are the primary limitation on the *Q* value of BICs, a challenge commonly encountered in the development of high-*Q* on-chip resonators [16], [17], [21].

Structural defects, which can be considered as perturbations introduced to photonic structures, can either deteriorate or enhance the optical properties of a structure [22]. Recent studies have highlighted that in systems with intentional structural defects, they can improve electromagnetic field enhancement [23] and localization [24] and stabilize topological states [25], [26], [27], [28]. In work [29], structural disorder leads to the emergence of a bound state in the continuum band in configurations involving multiple chains and layers. Novel strategies for achieving disorder-resistant ultrahigh *Q* factors using Brillouin zone folding in BIC metasurfaces have been proposed recently [30]. Moreover, a merging BIC was found to exhibit significantly reduced radiation losses compared to an isolated BIC within a similarly disordered metasurface [31]. Overall, the development and stability of resonant optical effects resistant to structural defects remain a key challenge in the contemporary nanophotonics research [23], [32], [33], [34], [35], [36].

Here, we present both experimental and theoretical analyses of the *Q* factor for BIC and guided leaky mode in a chain of Mie-resonant ceramic disks with structural disorder. We show that radiative *Q* factor decreases linearly with increasing disorder amplitude, in contrast to the one-dimensional periodic structure composed of two layers of dielectric rods considered in our previous work [34]. We verify our results experimentally in the radiofrequency (RF) range. In contrast to the optical range, RF experiments allow us to neglect the surface roughness of the structural elements and consider only their deviation from the initial position.

## 2 Regular periodic chain

### 2.1 Infinite chain

**Figure 1:**
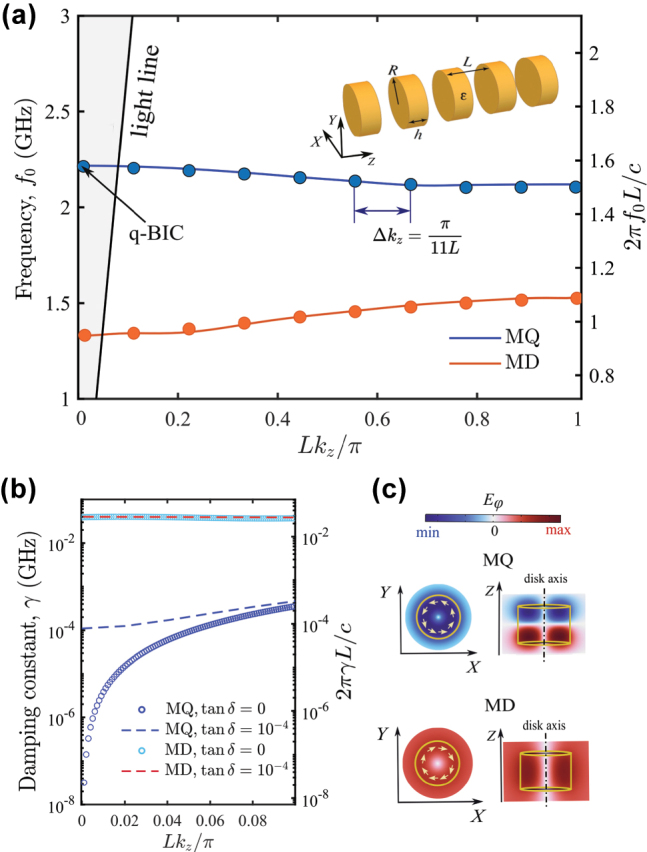
Eigenmode analysis. (a) Band diagram for the infinite chain of the ceramic disks. The circles show the eigenfrequencies of the finite chain consisting of 10 disks. The inset shows a 3D model of the chain. (b) Radiative losses dependencies for eigenmodes of the infinite chain in the presence and absence of loss. (c) Side and front views of the distribution of the azimuthal electric field of the MD and MQ eigenmodes.

Let us consider an infinite periodic chain of ceramic disks with period *L* (see the inset in Figure 1(a)). According to Bloch’s theorem, the electric field of an eigenmode in a coaxial structure can be written as:

(1) $$E\left(r,\phi ,z\right)=U_{m,k_{z}}\left(r,z\right)e^{\pm ik_{z}z\pm im\phi },$$

where *k*_*z*_ is the Bloch wave vector, *m* is the projection of the angular momentum (magnetic quantum number), and $U_{m,k_{z}}\left(r,z\right)$ is the periodic function of *z* with period *L*. The modes propagating in the +*z* and −*z* directions are degenerate, which leads to ± signs in the exponential. Similarly, due to the rotational symmetry, modes with ±*imϕ* are degenerate. The periodic Bloch amplitude can be expanded into a Fourier series:

(2) $$U_{m,k_{z}}\left(r,z\right)=\underset{n}{\sum }C_{m,k_{z}}^{n}\left(r\right)e^{i2\pi nz/L},$$

where $C_{m,k_{z}}^{n}\left(r\right)$ is the Fourier coefficient and *n* is an integer indicating the diffraction order. In the subwavelength regime, *λ* > *L*, we only have one open diffraction channel, i.e., only the zeroth Fourier coefficient $C_{m,k_{z}}^{0}\left(r\right)$ is nonzero and contributes to the far-field.

The modes with *m* = 0 have well distinctive polarizations, i.e., they can be divided into purely magnetic (TE) and electric (TM) types, while the modes with *m* ≠ 0 have a hybrid polarization [37]. Ought to this fact the modes with *m* = 0 can be symmetry-protected BICs at the Γ-point, while modes with *m* ≠ 0 can turn into a BIC only due to fine tuning parameters of the system. An additional advantage of the modes with *m* = 0 from the experimental point of view is that they can be selectively excited by the magnetic of electric probe antenna. Therefore, further we will focus only to the modes with *m* = 0. We will study two fundamental TE (magnetic) modes representing a chain of coupled magnetic dipoles (MD) and magnetic quadrupoles (MQ). The polarization of the modes is the following **E** = (0, *E*_*ϕ*_, 0) and **H** = (*H*_*r*_, 0, *H*_*z*_).

We start our study with numerical simulations of the eigenmodes of the infinite chain composed of ceramic disks with permittivity *ɛ* = 44, loss tangent tan*δ* = 10^−4^, disk radius *R* = 15 mm, disk height *h* = 20 mm, and chain period *L* = 34 mm. The fundamental magnetic Mie modes of the disk are magnetic dipole (MD) and magnetic quadrupole (MQ). Their field distribution is shown in Figure 1(c). Due to the coupling of the disks, the MD and MQ bands are formed. Figure 1(a) shows the band diagram of the infinite chain, where the bottom branch (orange line) corresponds to the MD mode and the top branch (blue line) – to the MQ mode. The complete band structure accounting other Mie resonances are shown in Figure S1. Although the MQ mode belongs to the radiative continuum, in fact, in the Γ point, this state is low-radiative with damping constant *γ* = 10^−4^ GHz in the lossy case. However, without material absorption, *γ* tends toward zero in the center of the Brillouin zone (Figure 1(b)). Taking into account the symmetry of the field distribution, one can conclude that it is a symmetry-protected BIC [38]. Due to large permittivity, modes are highly localized within the disks; therefore, the total *Q* factor of BIC is limited by the inverse value of tan*δ*, i.e., about 10,000. In contrast, the MD branch exhibits rather large radiative losses (orange line in Figure 1(b)), so it corresponds to the bright mode.

### 2.2 Finite chain

In the experiment, we deal with chains of a finite length. In this case, the ends of the chain play the role of partially reflecting mirrors and, thus, the chain can be considered a Fabry–Pérot resonator. As a result, for a chain of *N* scatterers, the continuous band of an infinite chain is replaced by a finite set of *N* Fabry–Pérot resonances at the frequencies corresponding to the quantized quasi-wave vector Δ*k*_*z*_ = *π*/[(*N* + 1)*L*] [39], [40]. Therefore, in a finite chain, a genuine BIC turns into a quasi-BIC with a finite *Q* factor. The resonant state with the highest *Q* factor has a quantized wave vector closest to the BIC one. For *N* = 10, in Figure 1(a), eigenfrequencies associated with MD and MQ branches are shown with circle markers, and quasi-BIC appear in the vicinity of the Γ point.

## 3 Effect of disorder

### 3.1 Numerical analysis

**Figure 2:**
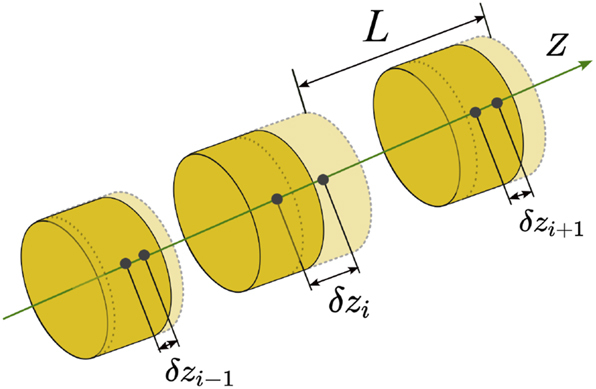
Schematic representation of a disordered chain of disks with period *L*. The disorder is induced by shifting the *z*-coordinates of the disks’ centers by *δz*_*i*_, where *i* is the number of the disk.

We investigate the effect of structural disorder in theory and then confirm it with experiments, considering consider finite chains composed of 10, 30, and 50 disks. We introduce uncorrelated disorder to the structure by shifting the coordinates of the disks’ centers by *δz*_*i*_ along the *z*-axis (Figure 2). The shift is given by

(3) $$\delta z_{i}=\varrho_{i}L\sigma ,$$

where *σ* is the disorder amplitude, *ϱ*_*i*_ is a random variable distributed uniformly within the interval [−1; 1], and *i* is the number of the disk. Thus, by applying *δz*_*i*_ to the *z*-coordinates of the disks’ centers, we provide a random shift and regulate its magnitude by changing *σ*. To analyze the evolution of the *Q* factor, we calculate the eigenmodes of the chain. In particular, for a given value of *σ*, an ensemble of a hundred randomly generated chains are examined. Among the obtained solutions, we select the fundamental Fabry–Pérot mode, i.e., the closest resonance to the Γ point, which could be associated with a quasi-BIC. For *N* = 10, the fundamental mode is quite easy to find even for a large *σ*. As the chain length increases, a more complex behavior is observed: (i) many defect modes with electromagnetic field localized in dimers or trimers (see the field distribution in the Section V of Supplementary Material) appear; (ii) there are two sets of similarly modulated Fabry–Pérot modes: the first, second, and so on caused by the field localization at the different ends of the chain. To distinguish proper eigenmodes and perform their statistical analysis, we have developed and implemented a postprocessing algorithm with manual control realized in MatLab software. The algorithm employs the 3-sigma rule for small values of *σ*. In ambiguous cases, we compared the frequency of a mode with the average values for the previous *σ* using the band diagram. We avoided defect modes with field localized on dimers and trimers and prioritized those whose field was distributed more uniformly and widely along the length of the chain. Due to the described difficulties, for long chains, we only considered *σ* up to 0.15.

**Figure 3:**
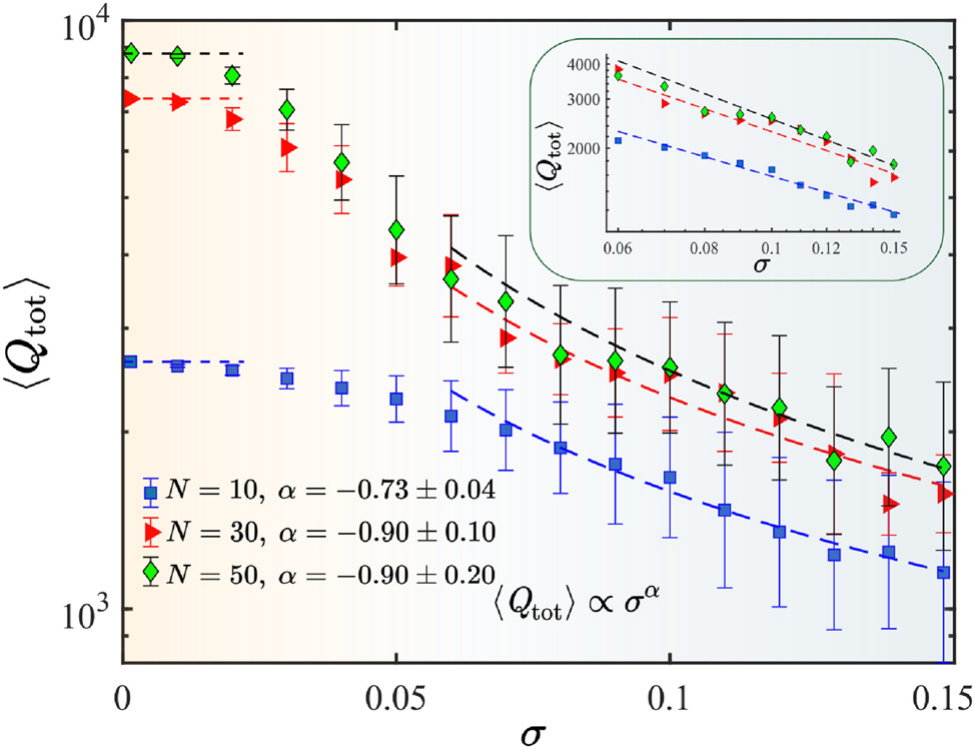
Total *Q* factor of the quasi-BIC depending on the disorder parameter *σ* for different numbers of disks in the chain *N*. We examined an ensemble of 100 chains for each value of disorder amplitude. The inset shows the *Q* factor dependencies in double logarithmic scale. With the background color, we distinguish the leading channel of losses. For small *σ*, the losses due to the finite size dominate; for large *σ*, structural disorder plays the main role.

As shown previously, due to the finite size of a structure, a nonradiative genuine BIC transforms into a quasi-BIC with a finite *Q* factor [40], [41]. In addition to radiative losses, we introduce material absorption as a tangent loss of tan*δ* = 10^−4^, estimated from Ref. [42]. For high-refractive index ceramics, in which the electromagnetic field is mainly concentrated inside the resonators, the maximum achievable value of *Q* is limited by the inverse loss tangent, i.e., 10^4^. For instance, in a lossless chain of 50 disks, the *Q* factor of a quasi-BIC is almost 10^5^, while in the lossy case, it is about 9,000 (see Figure 3, green markers). With increasing *N*, the *Q* factor approaches its limit of 10^4^ defined only by material absorption.

In general, the presence of disorder leads to the mixing of modes with the different wave vectors [34], [43]. For an infinitely long periodic chain, weak disorder leads to the slight change of the wave vector, which in turn provides a quadratic decline of radiative lifetime and the *Q* factor. This is due to the fact that, in the vicinity of the Γ point, the dependence of *Q* factor from wave vector is quadratic [38]. Moreover, a disorder introduced into the system leads to additional radiative losses. Thus, the overall *Q* factor is determined by

(4) $$\frac{1}{Q_{\text{tot}}}=\frac{1}{Q_{N}}+\frac{1}{Q_{\text{abs}}}+\frac{1}{Q_{\text{dis}}},$$

where losses due to the finite size, material absorption, and parasitic rescattering on structural disorder are summed. In the case of finite chain, among the observed loss mechanisms, the dominant one can be distinguished. While *σ* is small, the *Q* factor is approximately constant, and its value is determined by the losses stemming from the finite size and absorption [34]. For example, for an ordered chain with *N* = 10, *Q* is 2,600, see the blue markers in Figure 3. With increasing *σ*, the *Q* factor becomes sensitive to structural disorder. Starting from *σ* ≈ 0.02, the *Q* factor decreases notably. Once the disorder amplitude reaches a threshold of *σ* ≈ 0.05, its further increase results in a sublinear decay of the *Q* factor, indicating that the scattering effects due to the structural disorder dominate over those of finite size and absorption. In other words, as the disorder increases, reaching approximately several percent of the period, the intraband interactions enhance, resulting in mixing of modes and decreasing of the *Q* factor. In this case, as we show below, the *Q* factor decay law depends on multipolar origin of the BIC. Notably, the *Q* factor decay law tends to a linear function as the number of discs increases. In contrast to the obtained results, recently it has been shown that in a two-layered one-dimensional periodic structure composed of two arrays of Teflon dielectric rods, *Q* factor decreases quadratically with *σ* [34]. Since Teflon has a lower permittivity compared to ceramics, we have decided to check whether the permittivity value affects the *Q* factor decrease law, see Figure S4 in the Supplementary Material. We consider *ɛ* = 15 and *ɛ* = 6 and conclude that regardless of its value, *Q* decays linearly. For smaller values of *ɛ*, the field is localized weakly, and our method cannot define *Q* factor in the case of disorder properly. It is notable that in Ref. [34] the authors consider a q-BIC associated with a magnetic dipole mode (MD), whereas our study focuses on a magnetic quadrupole q-BIC (MQ). We have calculated the *Q* factor of a magnetic dipole q-BIC in a ceramic grating with the optical contrast and cross section similar to those of the chain, see Figure S9 in Supplementary Material. In addition to simulations, we have developed a theoretical model, see Section 4, and obtained the *Q* factor of the magnetic dipole mode in a chain of lossless circular rods with the same permittivity as for the disks (see Figure S10 Supplementary Material). As a result, we have obtained a near-quadratic decaying of the *Q* factor of the dipole q-BIC. Thus, within the specified range of permittivity 6 ≤ *ɛ* ≤ 44, the optical contrast has no impact on the law governing the decrease in the *Q* factor, i.e., the general sublinear behavior is maintained. Based on the comparison of q-BICs for MD and MQ modes, we can assume that the *Q* factor dependence on *σ* is related to the multipolar origin of the mode. It has been shown recently that in a periodic array with a regular asymmetry, the scaling law of the *Q* factor depends on the origin of the mode [44]. Moreover, disorder acts as an effective perturbation with respect to the wave vector, leading to a shift in the position of the q-BIC, which is originally located at the Γ point when *σ* = 0 (Figure 1(a)). According to the results of the article [45], the quality factor of the q-BIC for the different multipoles demonstrates distinct asymptotic behavior as a function of the wave vector upon deviation from the Γ point. Following these ideas, rather strong resistance of the quadrupole q-BIC compared to the dipole q-BIC can be attributed to the high field localization of the MQ mode, see Figure S11 in Supplementary Material. That is, unlike dipolar BICs, which show a quadratic decay of the *Q* factor due to symmetric perturbations near the Γ-point [34], [46], the MQ-BIC exhibits a linear decay, illustrating the influence of its multipolar nature and field localization properties.

For large *σ* values, the difference between the average *Q* factors of chains with different lengths starts to decline, and at *σ* > 0.15, it becomes negligible, manifesting spatial localization of the mode at a scale less than the length of the structure [34].

In addition to uniform distribution discussed above, we have investigated the normally distributed random displacements of the disks center of the lossless chain. In both cases, the *Q* factor decays almost linearly. More details are provided in Sections II and IV of the Supplementary Material. Although only longitudinal disorder is considered in this study, we expect that transverse disorder would destroy BIC more slowly than the longitudinal one since the distance between disks along *z* is fixed. Furthermore, the introduction of such disorder disrupts the system’s rotational symmetry. As a result, the evolution of the *Q* factor becomes more complex. However, these suggestions require more detailed investigation and are beyond the scope of the present work.

### 3.2 Experimental study of BIC and non-BIC modes

We considered a chain of 10 cylindrical resonators made of a low-loss microwave ceramic material based on the LaAlO_3_–CaTiO_3_ system, with nominal permittivity *ɛ* = 44, and the tangent loss tan*δ* = 1 × 10^−4^ at a frequency of 1 GHz, as previously described in Refs. [39], [40]. Each resonator has a diameter of 30 mm and a height of 20 mm. All the resonators have their axis aligned along a straight line. To control their positions along their common axis, a holder made of a microwave transparent material was designed and fabricated. As the holder material, Styrofoam with (*ɛ* ≈ 1.02 − 1.04) and negligible losses was chosen. Despite its very low weight, this material is rigid enough and has the sufficient rigidity to ensure precise placement of the resonators. A CNC milling machine was used to cut the holder out of a 5 cm thick foam slab. A cavity was cut out to place the disc resonators coaxially, with varying spacing between them.

**Figure 4:**
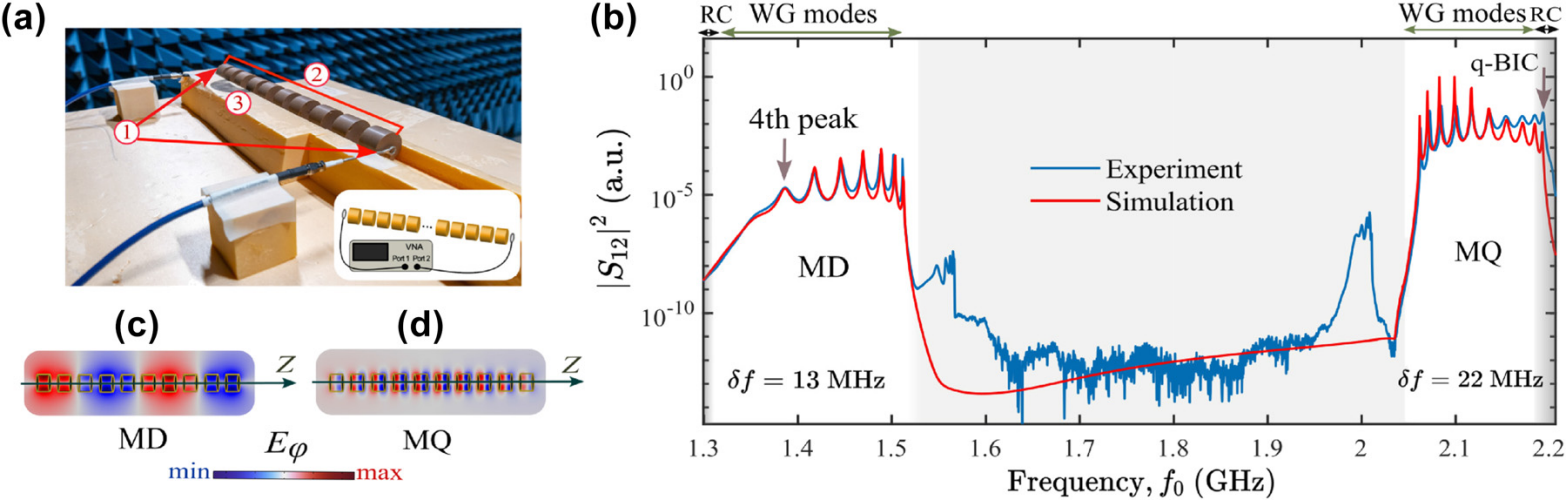
Experimental setup and transmittance spectrum of the finite chain. (a) Photo and scheme of the setup and samples. 1 – antennas, 2 – ceramic discs, 3 – foam holder. (b) Comparison of simulated (red curves) and experimental (blue curves) transmission spectrum of the 10-disk chain after taking into account the spectral shift *δf*. Panels (c) and (d) show the distribution of the electric field in the 10-disk chain for MD and MQ eigenmodes. Gray regions correspond to the radiation continuum, while the region of waveguide (WG) modes is indicated with the background color.

To measure the transmission and reflection coefficients of the structure, a Keysight (Agilent) E8362C Vector Network Analyzer (VNA) was used with a pair of magnetic dipole antennas connected to the VNA ports, see Figure 4 (a). The antennas’ axes were aligned with the axis of the disk chain. The size of the magnetic antennas was chosen so that their own resonances (at about 5 GHz) were high above the frequency band of interest (2–3 GHz). Therefore, in the frequency band of 2–3 GHz, their parameters are almost independent of the frequency. Thus, the measured complex values of *S*_21_ of this setup are proportional to the transmission and reflection coefficients of the resonator chain. We needed a large number of frequency samples to ensure sufficient resolution and carefully observe high-*Q* modes in the transmission spectrum. We decided to take measurements at twenty points in the interval *σ* ∈ [0; 0.2] with an increment of 0.01. For each disorder level *σ*, we performed 10 measurements, corresponding to 10 independent realizations of the disorder configuration. Then, for each measurement, we plotted the modulus of the vector *S*_12_ depending on the signal frequency and used these plots to determine the peak in the vicinity of the assumed resonant frequency *f*_0_. It is important to note that, regardless of the level of disorder, we observe a resonant transmission through the chain. Examples of transmittance spectra in the presence of disorder are provided in Figure S8 in Supplementary Material.

A representative measured transmission spectrum for a chain of 10 disks placed equidistantly is plotted in Figure 4(b). We observe two resonant transmission bands corresponding to the MD and MQ modes. The resonances lying in the unshaded area correspond to the waveguide modes, and those in the gray area are leaky modes coupled to the radiation continuum. The simulated spectra show good agreement with our experiment, but there are frequency shifts *δf* = 0.013 GHz and *δf* = 0.022 GHz for the MD and MQ modes, respectively. Two anomalous peaks within the band gap observed at about 1.55 and 2 GHz in experiment correspond to modes with *m* ≠ 0, compare with Figure S1 in Supplementary Material. These modes are excited due to imperfect alignment of antennas.

**Figure 5:**
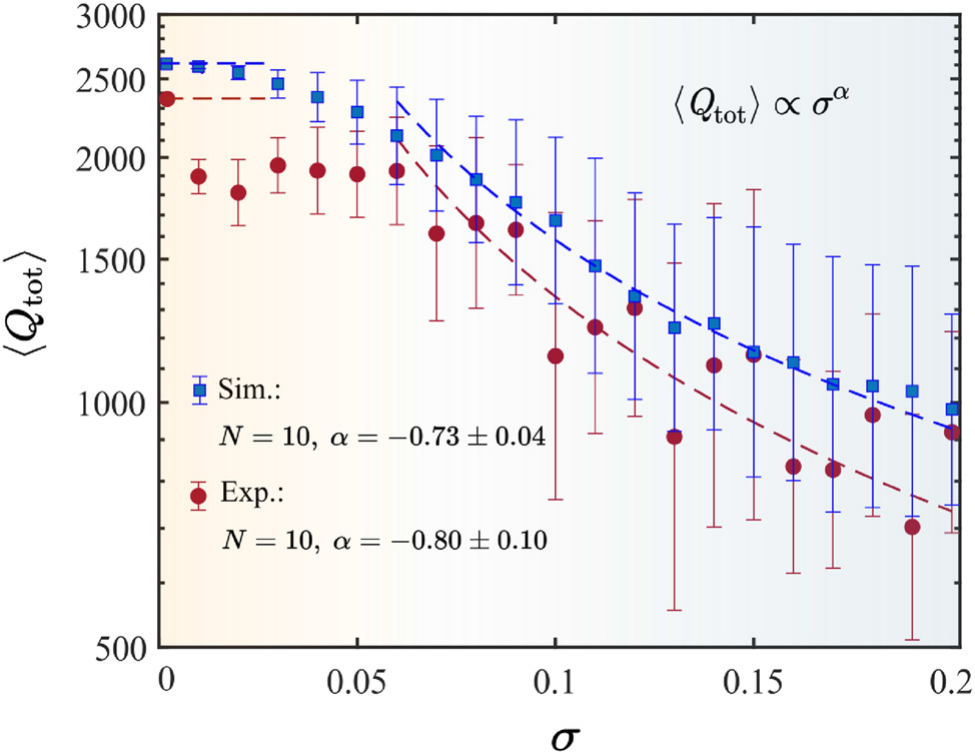
Theoretical and experimental dependencies of the total *Q* factor of a quasi-BIC on the disorder parameter. The markers show the average *Q*, while bars depict the standard deviation. In transmittance measurements, the chain was reassembled 10 times for each disorder amplitude *σ*. In simulations, an ensemble of 100 chains was studied.

First, we focus on the MQ mode turning into BIC. We observe 10 peaks in the spectrum, as it should be for a chain of 10 disks. The last peak, i.e., the highest-frequency one, corresponds to a quasi-BIC, and its field distribution is plotted in Figure 4(c) and (d). The loaded *Q* factor is extracted as *Q* = *f*_0_/Δ*f*, where Δ*f* is the full width of the local maximum in the transmission spectrum at frequency *f*_0_. The width and resonant frequency are extracted by fitting the transmission spectrum with the Fano formula [47]. The calculated and extracted *Q* factors and the approximation of the obtained dependencies are plotted in Figure 5. As we mentioned above, two regions can be discerned in terms of the dominant loss mechanism. At low *σ*, *Q* factor is mainly determined by the material absorption and radiative losses due to the finite size of the structure. However, the loaded *Q* is lower than the calculated one by ∼700 . It can be explained by coupling to antennas and parasitic scattering due to noncoaxial arrangement of the disks in the chain [40]. As the disorder amplitude increases, the difference becomes less significant, and both theoretical and experimental *Q* factors decay linearly with respect to *σ*.

**Figure 6:**
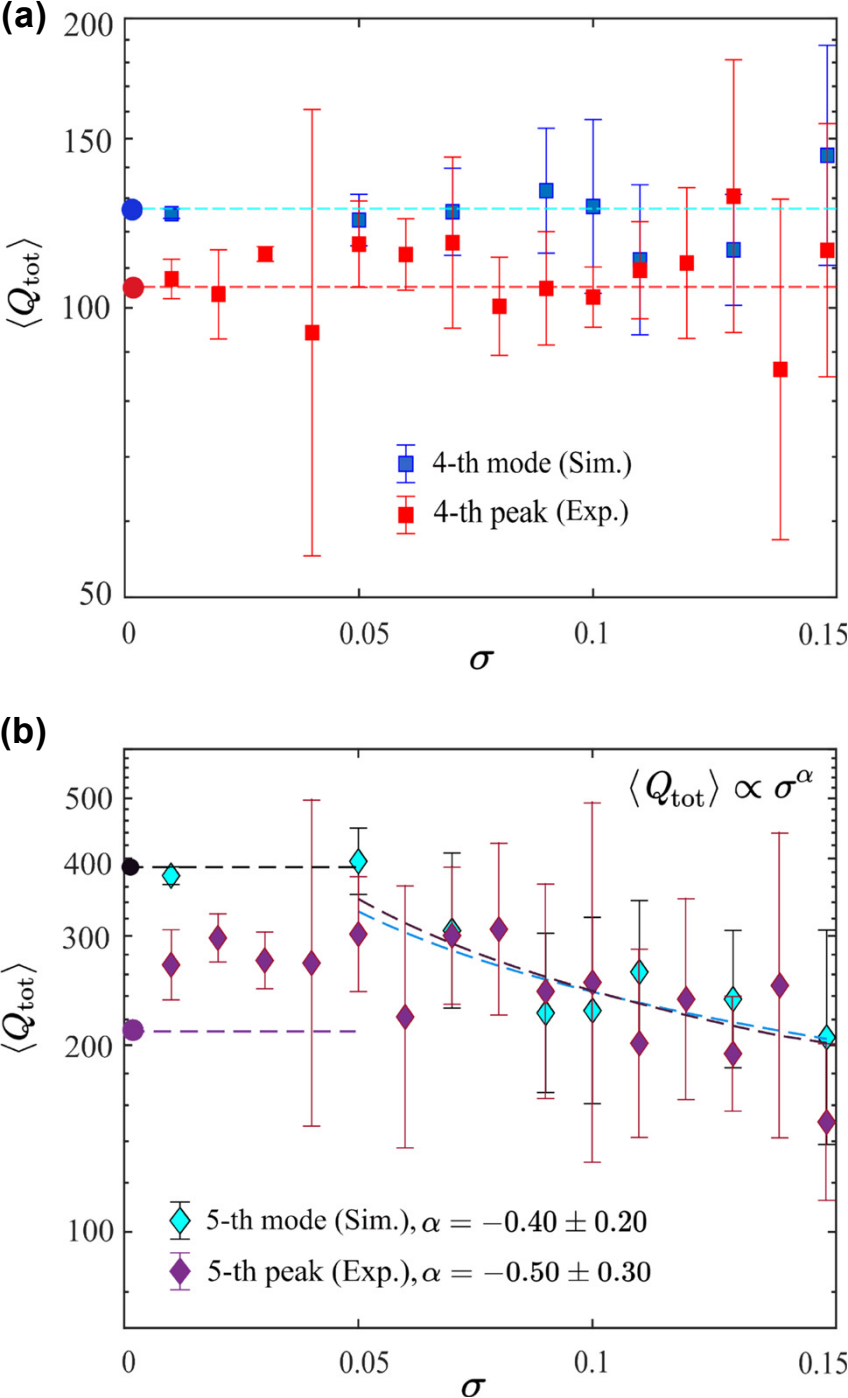
Total *Q* factor dependencies from disorder parameter for leaky mode. Results on panel (a) corresponds to the 4th peak, while in panel (b), the fitting results for 5th peak are listed. In the experiment, 5 different measurements were made for one value of disorder, while we consider 10 different chains during simulation.

Next, we consider the MD mode, whose spectrum is shown in Figure 4(b). Contrary to the MQ mode, here, we see 7 distinct peaks instead of 10. The other three peaks cannot be determined even in simulation because of their low *Q* factor. The peaks merge with the background or the 4th peak since its shape has notable asymmetry. To analyze robustness of the MD mode against structural disorder, we measure transmittance for a chain with varying amplitude of disorder *σ* ≤ 0.15. Then we extract the *Q* factors of the 4th and 5th peaks and compare them with the predictions from simulation of eigenmodes, see Figure 6. In the case *σ* = 0, the first mode corresponding to the peak with the lowest frequency has a low *Q* factor. The introduction of disorder causes this low *Q* resonance to mix with neighboring modes, resulting in a constant value of the average quality factor. Additionally, due to the limitations of the numerical method, it is not possible to determine this trend more precisely. Other modes have a higher *Q* factor; hence, we can observe the effect of disorder. Specifically, the *Q* factor of the 5th peak decays differently from that observed for the MD quasi-BIC from Figure S10 of Supplementary Material and the extracted exponent is ∼0.5 , indicating a sublinear decay (Figure 6(b)). This results from the complex interaction of it with neighboring modes, which may have either higher or lower *Q* factors.

Although our experimental platform is implemented in the microwave regime, the observed linear decay trend of the *Q* factor is expected to appear in optical systems as well, particularly in dielectric structures supporting high-quality multipolar resonances. This is in line with recent optical-domain studies where multipolar BICs show enhanced robustness to structural disorder (see Ref. [44]).

## 4 Analytical model

Here, we develop an analytical model that accounts for pairwise interactions between all resonators in the array. This model enables the analysis of the complex eigenfrequencies in a disordered array, as it incorporates the dependence of the coupling constant on the distance between the resonators.

Due to the interaction between Mie modes of the disks, resonant transmission occurs. It is clearly seen from Figure 4(b) that the signal drops dramatically for resonances above the light line. It happens due to the coupling to the radiation continuum. All this can be described in terms of coupled mode theory [48].

It is well known that the mode amplitude *ψ* in a system of *N* resonators can be described by the differential equation as follows [48], [49]:

(5) $$i\dot{|\psi 〉}=\hat{M}|\psi 〉,$$

with matrix elements $\hat{M}$ .

After the substitution of *ψ* = *ψ*_0_ *e* ^*iωt*^, one can get the eigenvalue problem for the matrix $\hat{M}$ :

(6) $$\det\left(\hat{M}-\omega \hat{I}\right)=0.$$

**Figure 7:**
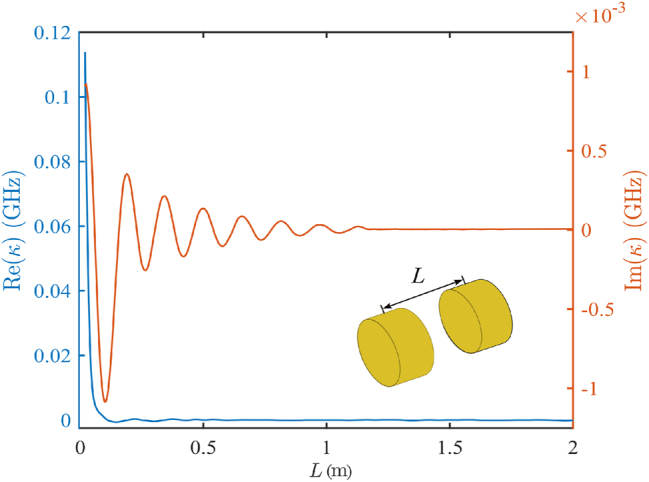
Dependence of coupling coefficient from the distance between two disks. The values are obtained by Eq. (8).

Matrix $\hat{M}$ has a form:

(7) $$M_{i,j}=\Omega \delta_{i,i}+\kappa_{i,j}\left(1-\delta_{i,j}\right),$$

where Ω is the complex resonant frequency of a single resonator, *κ*_*i*,*j*_ is the complex coupling coefficient, which depends on the distance between the *i*-th and *j*-th resonators in the chain, and *δ*_*i*,*j*_ is the Kronecker delta. Here, we assume that the imaginary part of Ω is only responsible for the coupling to the radiation continuum due to the absence of the material absorption. In our case, the diagonal elements of the matrix $\hat{M}$ are set to the eigenfrequency of the quadrupole mode Ω = 2.141 + 0.001*i* GHz. Note that we take into account the interaction between all the disks in the chain, i.e., we consider all off-diagonal terms of the coupling matrix. To analyze the coupling coefficients in the case of disorder, we simulated two lossless disks with varying distance *L* using COMSOL Multiphysics and obtained the frequencies of the symmetric and antisymmetric eigenmodes. Finally, the complex coupling coefficient can be found as follows [41]:

(8) $$\kappa =\frac{\Omega_{a}-\Omega_{s}}{2},$$

where the indexes *i*, *j* are omitted for convenience. The computed dependence of the complex coupling coefficient on the random distances between the disks *L* is shown in Figure 7.

**Figure 8:**
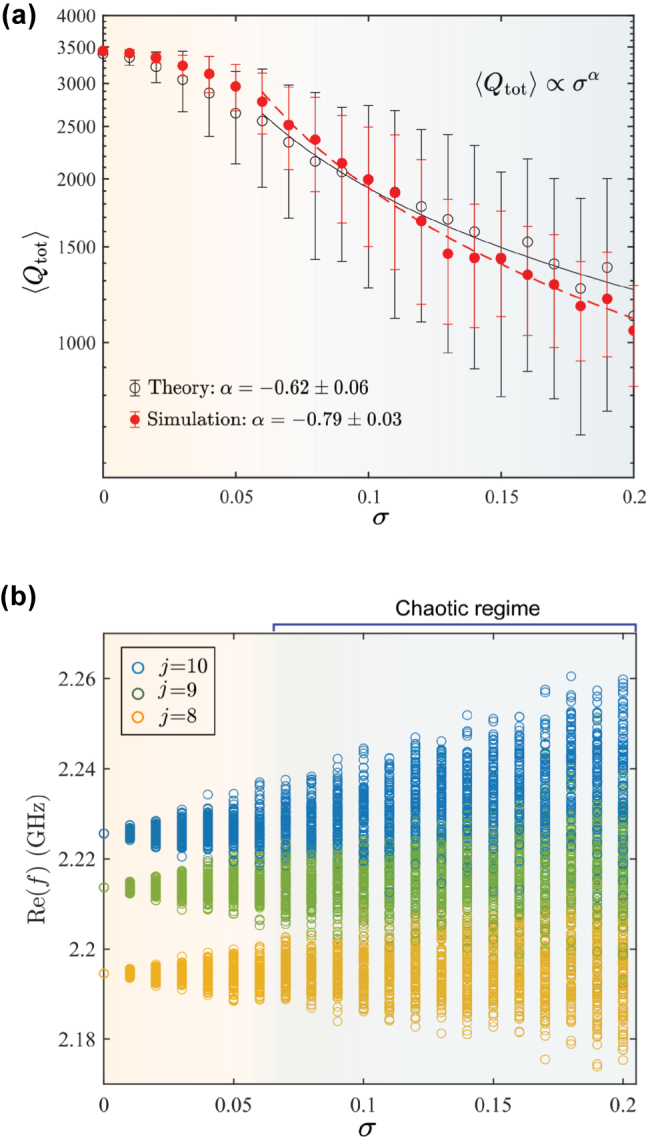
Analytical results. (a) Comparison of *Q* factor obtained from the solution of eigenvalue problem and simulation for the lossless chain of 10 disks. (b) Behavior of the real part of the 8th, 9th, and 10th eigenfrequencies as a function of the disorder parameter *σ* for 100 realizations. *j* denotes the index of the eigenfrequency. The background indicate the dominant loss mechanism: finite-size effects for weak disorder, and structural fluctuation for *σ* ≳ 0.5. In addition, these colors distinguishes the chaotic regime, where the eigenfrequencies begin to mix, from regular unmixed one.

The effective Hamiltonian $\hat{M}$ generated for an ensemble of one hundred random samples at the specified amplitude of disorder *σ* is employed to calculate the eigenmode spectrum of the system. The *Q* factor of the quasi-BIC for the chain of 10 lossless disks, calculated via theoretical model and simulation, is shown in Figure 8(a). The approximation is also presented for comparison. It can be observed that the results are in a good agreement, although the standard deviation is larger than that obtained from COMSOL Multiphysics simulations. This discrepancy can be attributed to the absence of an automatic filtering mechanism for selecting meaningful solutions in the theoretical approach, a feature that is available in COMSOL Multiphysics through mode profile selection. As it will be discussed further, for a larger value of *σ*, we observe mixing of 10th and 9th eigenmodes. In order to distinguish the quasi-BIC, we employed either the maximum real part of the frequency filter or the maximum *Q* factor filter. However, the two filtering algorithms yield the same approximation of *Q*(*σ*). Finally, both the theoretical approach and the simulation exhibit a consistent sublinear dependence of the *Q* factor on the disorder amplitude.

Furthermore, we examine the behavior of the fundamental mode and its two neighboring modes in relation to the disorder parameter. As illustrated in Figure 8(b), for values of *σ* below approximately 0.05, these modes remain distinct. However, as the disorder increases, a chaotic regime emerges, and the fundamental mode (*j* = 10) predominantly mixes with the next Fabry–Pérot mode (*j* = 9). This provides an explanation for the substantial decrease of the *Q* factor, for *σ* exceeding the threshold value, as demonstrated in both the theoretical and experimental results, see Figure 5.

## 5 Anderson localization

Anderson localization, characterized by the emergence of localized states, is a remarkable phenomenon observed in disordered systems [43], [50], [51]. It has been well studied in various physical systems, especially in 1D photonic structures [52], [53], [54]. In general, disorder disrupts the effective transport of energy through the system, leading to suppressed transmission efficiency [55]. In the case of finite structure, the localization effect occurs when the field becomes confined to a region smaller than the system’s characteristic size [56]. This results in the formation of localized states for which the transmitted field decays along the chain [43].

**Figure 9:**
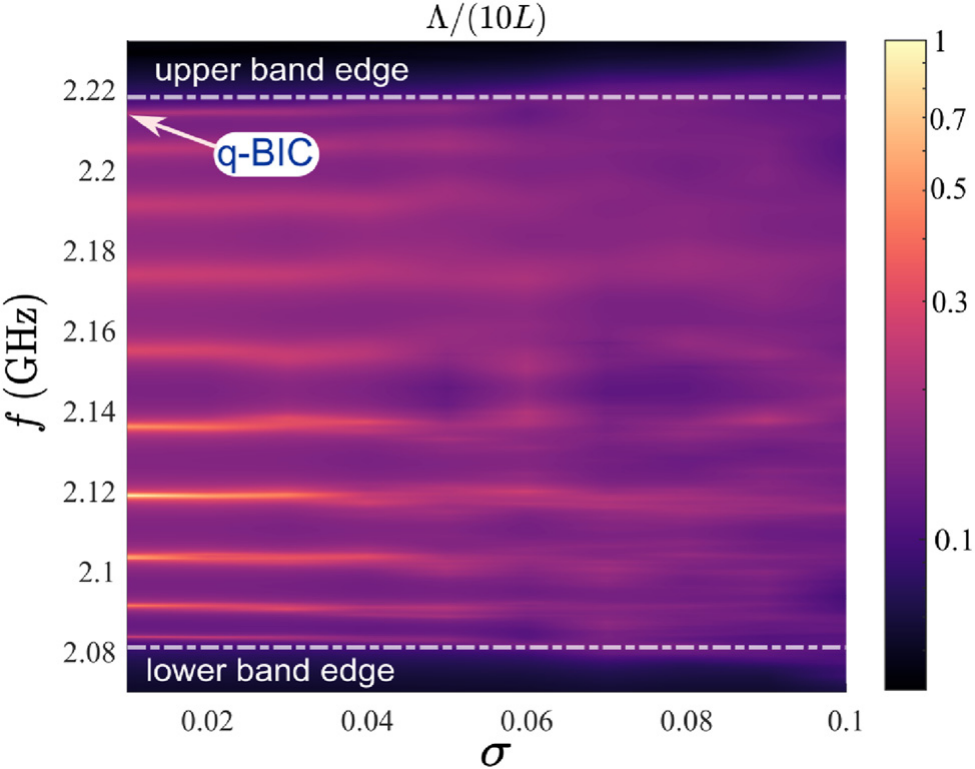
Numerically calculated localization length for the chain of 10 disks. The map highlights the upper and lower band edges and indicates the frequency of the q-BIC.

Here, we examine the influence of disorder on the transmitted field and the emergence of localized states in a disordered chain. For this, we calculated the evolution of a *localization length* Λ determined by transmittance *T* as follows [57]:

(9) $$\frac{1}{\Lambda }=-\frac{\left\langle \ln T\right\rangle }{NL},$$

where *N* in our case is equal to 10. Figure 9 shows the localization length as a function of *σ*. It is possible to distinguish 10 bright lines, which correspond to the 10 Fabry–Pérot modes. Among them, quasi-BIC is observed at the highest frequency. Moreover, the behavior of localization length indicates the presence of two regimes (Figure 9). Namely, at small *σ*, the resonant modes are well separated, while as the disorder increases, they start to mix. At the first regime, the localization length is comparable to the chain length or longer. The localization length is physically limited by both the finite chain size and the presence of nonzero material absorption. At the second regime, the localization length decreases dramatically, indicating the emergence of Anderson-like localization [43], [51], [57]. This, in turn, leads to the appearance of the spatial localized field along the disordered chain [58]. This behavior of the modes is the same as shown in Figure 8(b).

Furthermore, the localization length assumes small but nonzero in the vicinity of the band edge within the band gap (Figure 9). The band gap facilitates the formation of a highly localized field in a small region of the disordered system [57]. However, it is important to note that, irrespective of the level of disorder, both the simulation and the experiment demonstrate a resonant transmission through the chain, as illustrated in Figure S13 in the Supplementary Material. This fact serves to prove that our investigation is focused on the destroying of BIC rather than the spatial localization of the field in disordered media. In addition, while BICs arise due to the symmetry of modes in periodic systems, Anderson localization occurs as a result of strong disorder. In our system, increased disorder leads to mode mixing and a transition from extended quasi-BIC states to localized states, forming a bridge between the two regimes.

## 6 Conclusions

In conclusion, we have studied how uncorrelated structural disorder affects a symmetry-protected at-Γ BIC and a non-BIC mode in a one-dimensional periodic array composed of ceramic disks. In the experiment, we have selectively excited magnetic quadrupole and magnetic dipole modes with zero orbital angular momentum and measured the transmission spectra using coaxially placed loop antennas. We have extracted the *Q* factor from the experimental data for arrays with 10 disks, revealing different asymptotic depends of the *Q* factor on the disorder amplitude for the symmetry-protected BIC and non-BIC modes, respectively. Moreover, coupled mode theory predicts a sublinear decay of the *Q* factor, which is corroborated by both our numerical simulations and experimental results. The behavior of the *Q* factor for the magnetic dipole mode has been examined, and a well-known quadratic decay has been revealed. Therefore, we can conclude that, in the case of strongly disordered finite chain, where neighboring modes start to mix and Anderson localization appears, the intraband interaction became significant, and the *Q* factor decreasing depends on the multipole origin of the quasi-BIC. Moreover, we found that in a disordered finite chain, the quality factor behavior depends on the resonance order and its interaction with neighboring modes, which may have either high or low *Q* factors. Additionally, theoretical analysis predicts the range of disorder amplitude at which the chaotic regime emerges, which aligns with the estimated value from the simulations.

## Supplementary Material

Supplementary Material Details
