# Supplementary material for: Bound states in the continuum in a chain of coupled Mie resonators with structural disorder: theory and experiment
Source: Nanophotonics. 2025 Sep 17;14(19):3133–43. doi: 10.1515/nanoph-2025-0225 (PMC12455287; doi:10.1515/nanoph-2025-0225)
Supplement: Supplementary file 1 — Supplementary Material Details [file j_nanoph-2025-0225_suppl_001.pdf]

Supplementary Material
for
"Bound states in the continuum in a chain of coupled ceramic disks with structural disorder: theory and experiment "

I. BAND DIAGRAMS

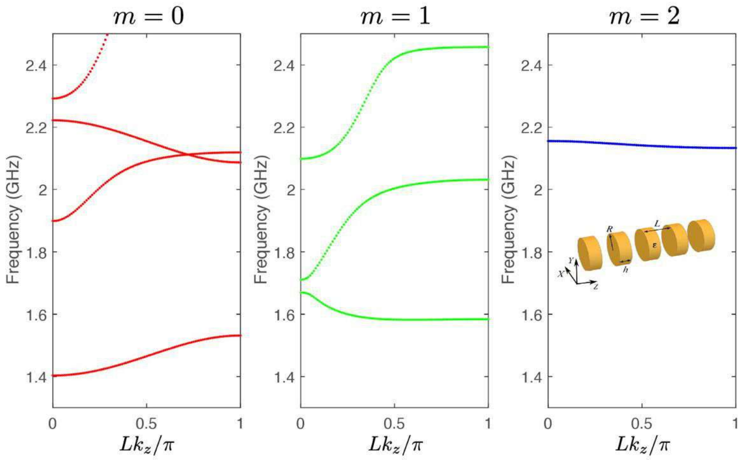

FIG. S1. Band diagrams for $m = 0, 1, 2$ in the frequency range of interest.

II. DISTRIBUTION OF QUALITY FACTOR

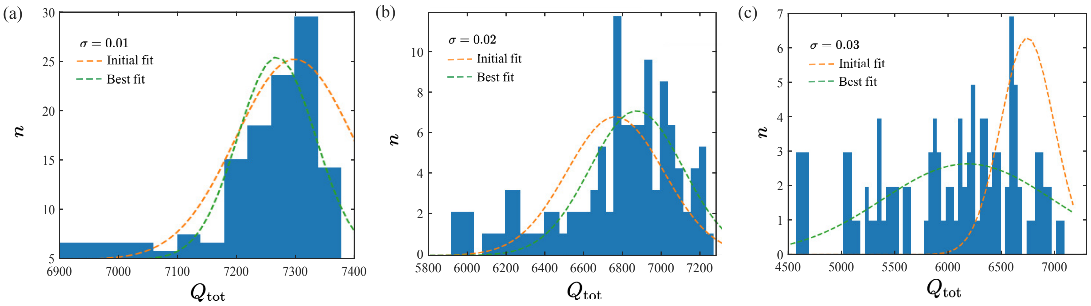

FIG. S2. Distribution of the total Q factor and its approximation by the normally distributed function. The chain is composed of 30 disks.

As it is shown in Fig. S2, with σ increasing, the maximum value of $Q_{\rm tot}$ changes and its distribution becomes more asymmetric. In contrast, for small values of σ, the asymmetry of the quality factor distribution from realizations remains low, indicating near-symmetric distribution. Since our numerical simulations are limited to 100 realizations, we decided to employ the CMT model to generate a larger number of different chains for a given level of disorder. Thus, we computed 10000 realizations of a chain with 10 disks, enabling a more precise observation of the asymmetric distribution of the quality factor [Fig. S13]. A rigorous mathematical proof of this phenomenon requires additional investigation and it's beyond the scope of the our work.

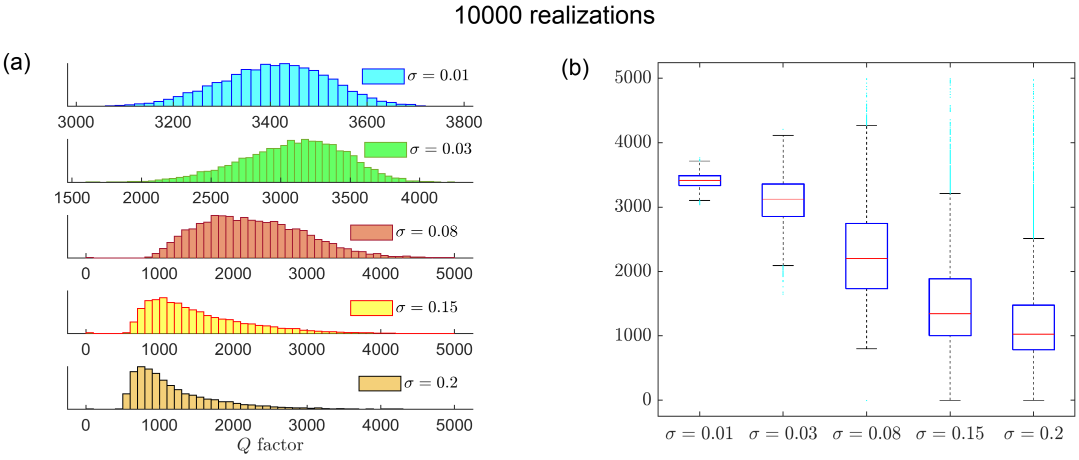

FIG. S3. Analytically obtained distribution of the Q factor from 10000 realizations for a chain of 10 disks at fixed values of disorder. Panel (a) shows the histogram of the Q factor, while panel (b) displays the corresponding boxplot. The results indicate that for smaller disorder values, the Q factor distribution is approximately symmetric. However, as the level of disorder increases, the distribution becomes increasingly asymmetric.

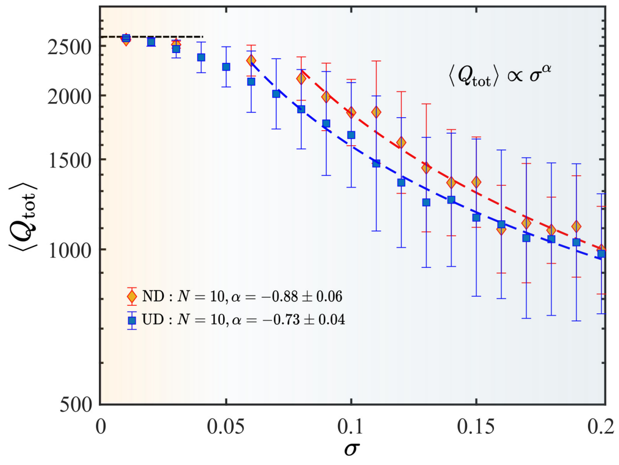

FIG. S4. Dependencies of the average value of total Q factor from disorder parameter for normal (ND) and uniform (UD) distributions random coordinates of the disk's center.

Next, we checked whether the distribution of a random shift of disks' centers affects the average value of Q factor. We compared the case of normal and uniform distribution of random shifts. For the small values of σ, there is no difference between normal (ND) and uniform distributions (UD) coordinates (Fig. S4). In contrast, with the σ increasing, obtained values of Q become slightly different. However, this difference do not change much the decay law. It should be noted that we studied the ensemble of 10 chains, so some discrepancy can appear due to the small amount of chains. For the larger values of σ, the behavior of the average of $Q_{\rm tot}$ in both case is the same.

III. VARIOUS VALUES OF DIELECTRIC PERMITTIVITY

In order to check whether the decrease of the dielectric permittivity ε of disks affects the decay law $Q \sim \sigma^{-1}$, we provide the similar series of simulations for lower values of ε then in the main text. With COMSOL Multiphysics and MatLab software, we analyse ensembles of 10 chains per each value of amplitude σ. The chain is composed of 10 disks. Figure S5(b) shows average Q factor for ε equals to 6 and 15. The lowest value of ε was chosen based on the assumption that eigenmodes should be spatially localised and do not interact with the perfectly matched (PML) layers surrounding simulation area even in the presence of disorder, see Fig. S5(a).

Although the slope of both lines is close to 1 and deviate from the quadratic low, the exact values of the α differ

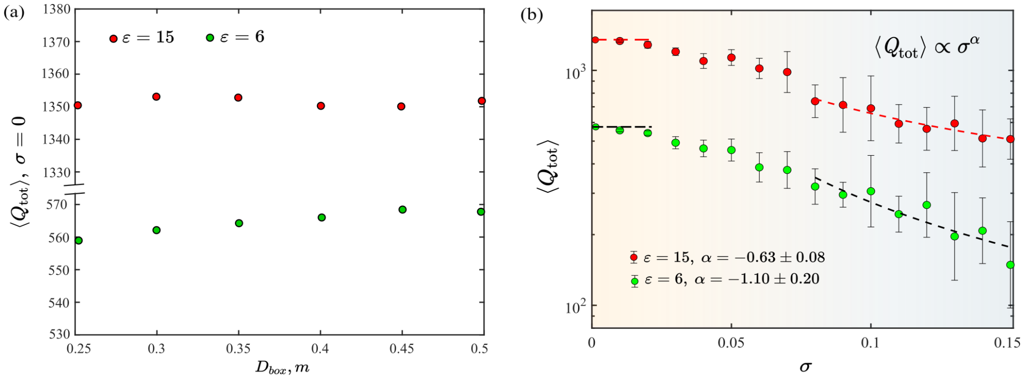

FIG. S5. (a) Total Q factor versus the box size D_{box} for different values of disks permittivity ε in the absence of disorder. (b) Dependencies of the average value of total Q factor from the disorder parameter for $\varepsilon = 15$ and $\varepsilon = 6$. During the simulation ensemble of 10 chains were studied.

between the $\varepsilon = 6$ and $\varepsilon = 15$ [Fig. S5(b)]. This difference indicates that α depends on ε. However, uncovering the precise relationship between ε and α remains a challenging question. We also note that the obtained results are based on a finite ensemble of randomly disordered structures. The extracted exponents are therefore naturally subject to statistical fluctuations and may be sensitive to the identification of modes, especially in regimes where disorder-induced mixing is significant.

IV. INFLUENCE OF THE MATERIAL LOSSES ON QUALITY FACTOR IN THE DISORDERED CHAIN

In order to observe the effect of the material losses on the Q factor behavior, we studied modes of chain of 10 disks. For a particular value of σ, we examined 100 randomly generated chains. The obtained results are depicted in Fig. S6. Obviously, at any value of σ, Q factor is larger for the lossless chains then for the lossy ones. However, in the lossless case, Q factor decreases following the similar decay law as in the lossy case.

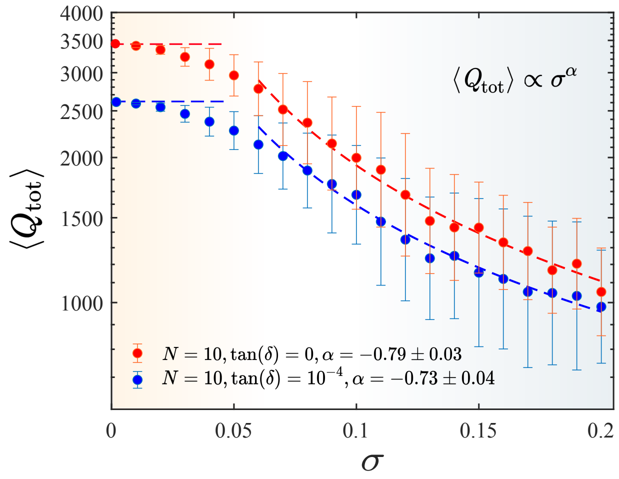

FIG. S6. Comparison of average Q factor dependencies from the disorder parameter at the 10-disk chain in case of presence and absence of material losses.

V. ELECTROMAGNETIC FIELD DISTRIBUTION IN THE CHAIN WITH DISORDER

The increasing of the chain's length leads to the more complex behavior of the mode. Figure S7 shows the appearance of localised defect modes in dimers or trimers in the 30-disk chain. Moreover, as amplitude of the disorder increase,

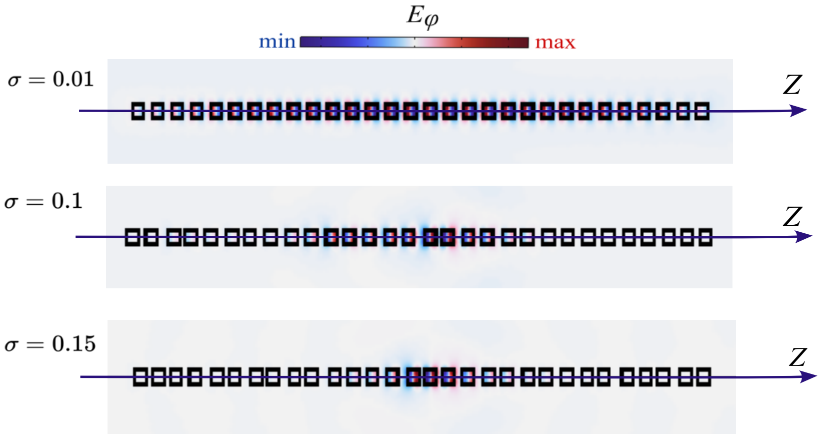

FIG. S7. Electric field distribution in the 30-disk chain for the different value of disorder σ. While the top panel shows the fundamental Fabry-Perot mode, the bottom ones show the defect modes.

these localised modes became narrowly distributed along the length of the chain. During simulations, we exclude the defect modes. Outside the disk, the field of a quasi-BIC decays exponentially with the distance. This behavior of the mode is shown in Fig.S8. The simulation results (black dots) indicate that for the distance $r/R > 4$, the field distribution deviate from the approximation curve. Moreover, when $r/R \geq 7$, electric field became constant. This behavior of the field is related to the limits of the accuracy of simulation method.

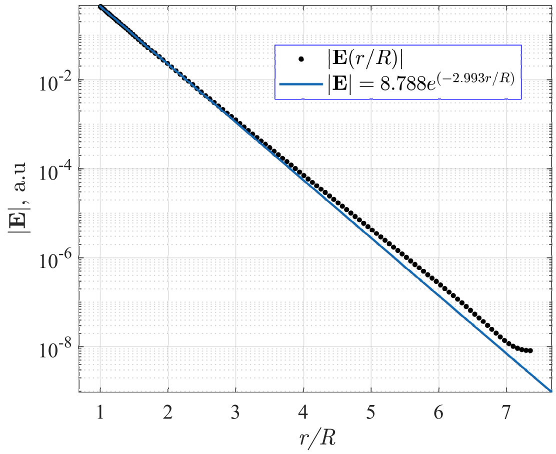

FIG. S8. Normalized BIC's field dependence from the distance outside the disk in the case of infinite periodic chain.

VI. TRANSMISSION SPECTRA OF DISORDERED CHAINS

Undoubtedly, the presence of the disorder affects the spectral response of the system. It can be clearly seen in Fig.S9, which illustrates the evolution of the transmittance spectrum with the disorder amplitude. With the σ increasing, the amplitude of the response decreases, while level of noise increases. The bands appeared at about 3 GHz vanishes. However, we observe the modes of interest at the same frequencies regardless the value of σ.

VII. MULTIPOLAR ORIGIN OF Q FACTOR DEPENDENCIES FROM THE DISORDER

In the previous research, it was shown that the Q factor decays quadratically with the disorder parameter [S1]. The authors studied q-BICs for the dipole modes of the double array of dielectric rods. Since here we consider a

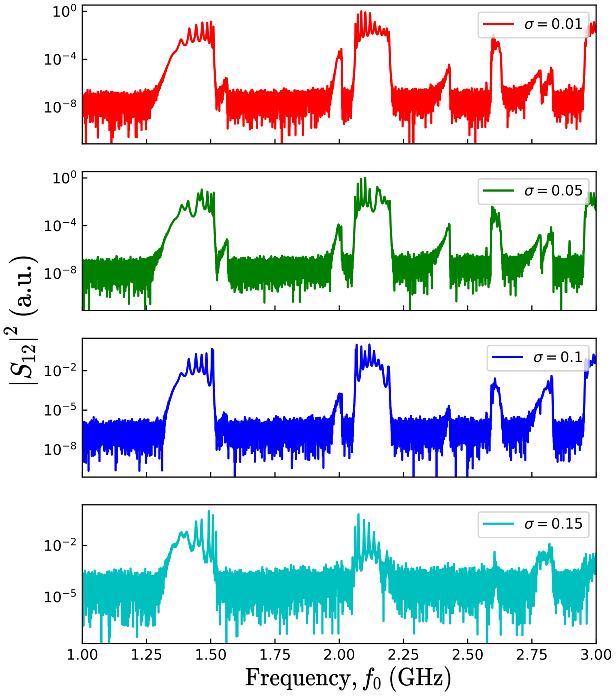

FIG. S9. Measured transmission spectrum of the 10-disk chain with different value of disorder parameter σ.

quadrupole q-BIC and obtain $Q \sim \sigma^{-1}$, we suppose that the Q factor dependence from σ is related to the multipolar origin of q-BICs. In order to test this hypothesis, we calculate Q for the dipole q-BICs in the finite array of infinitely long ceramic rectangular rods. The height of the rods is equal to the disk's diameter, while the width is similar to the disk's height. Here, we have chosen a grating, since dipole modes of the chain considered in the main text does not support BICs due to the symmetry requirements, see Fig.1(c). The finite array consists of 50 rods. For a given value of σ, we calculated 10 randomly generated finite arrays. Figure S10 shows the behavior of total Q factor from σ for two q-BICs. One can see that the Q of the dipole (MD) q-BIC decays faster then the quadrupole q-BICs (MQ). We suppose that some discrepancy between expected quadratic decay law and obtained $Q \sim \sigma^{-1.5}$ can appear due to the small amount of chains.

In order to verify the obtained results, we apply the Coupled Mode Theory (CMT) to both dipole and quadrupole modes. A detailed description of the applied theory is provided in the main text. In this analysis, we consider a lossless finite array of 10 circular rods with the same permittivity as that of the disks. The period of the array is 600 nm, while the radius is equal to 225 nm. In the case of an infinite periodic array, we found that a magnetic dipole mode exhibited a at-Γ BIC at a frequency of 121.4 THz. As it shown at Fig. S11, the decay of the Q factor is similar to that observed value in the simulation results. Consequently, it can be concluded that the decay of the Q factor is independent of the geometry and can be explained in terms of multipoles.

Obviously, Q factor depends on the localisation of the field in the resonator. Figure S12 shows the field behaviour along the direction of periodicity z within a unit cell. We examine magnetic quadrupole (MQ) and dipole (MD) modes of the grating and chain of disks. One can see that the field associated with BIC decays linearly, while the leaky MD mode of the chain shows nonlinear dependence. Moreover, BICs associated with MQ modes decays faster manifesting about strong localization compared to MD-BIC in the grating.

VIII. DEPENDENCIES OF TOTAL Q FACTOR DECAY LAW FROM THE CHAIN LENGTH

In the main text from the simulation results, we conclude that with the increment of the chain length, the total Q factor dependencies from the disorder are close to $Q_{tot} \sim \sigma^{-1}$. In order to verify this, we theoretically calculate the Q_{tot} for $N = 30$ and $N = 50$ lossless disks. As it is shown at S13, the coefficient of total Q factor decay from the disorder amplitude is close to the $\alpha = 1$.

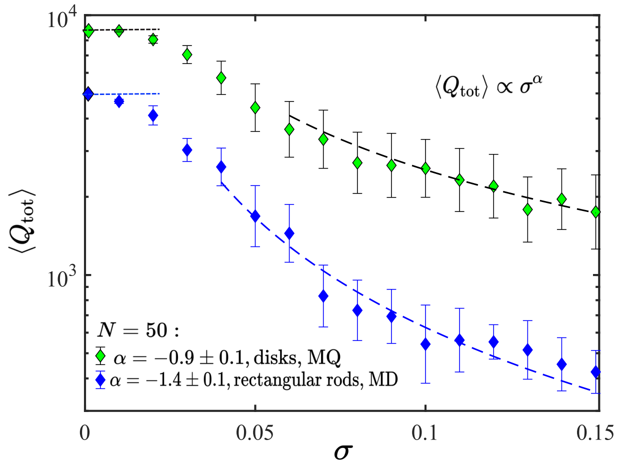

FIG. S10. Dependencies of the average value of total Q factor from disorder parameter for quadrupole (MQ) and dipole (MD) q-BICs. The quadrupole q-BIC is observed for the chain of disks, while the dipole q-BIC appear in the finite array of the rods with rectangular cross-section. The number of disks and rods is equal to 50.

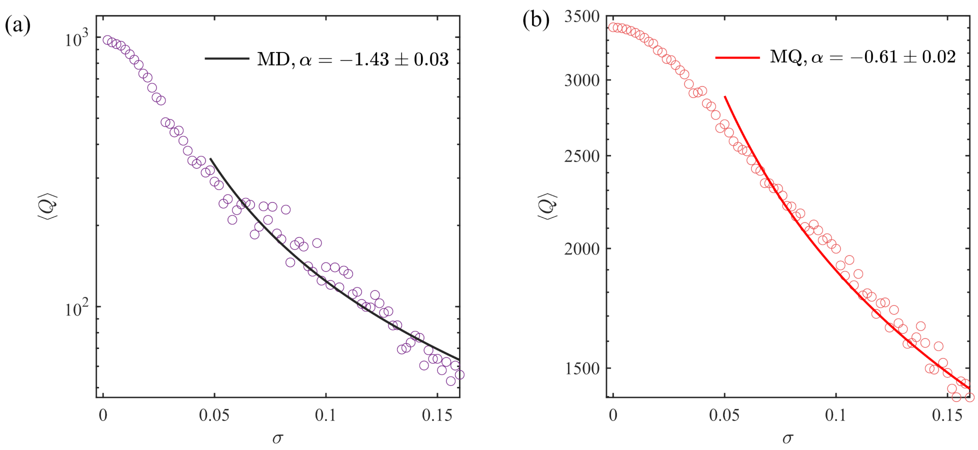

FIG. S11. Theoretically obtained dependencies of the mean value of the total Q factor from the disorder parameter (a) for a dipole mode (MD) in a finite chain of 10-circular rods and (b) for a quadrupole mode (MQ) in a chain of lossless 10 discs. The values averaged over 300 realizations.

IX. LOCALISATION LENGTH

Figure 9 from the main text demonstrates that the mixing of eigenfrequencies is facilitated by the emergence of localized states. A key parameter characterizing these localized states is the localization length. Physically, the localization length can be interpreted as the decay length of the transmittance outside the chain, therefore it can be found as:

$$\frac{1}{\Lambda} = -\lim_{N\to\infty} \frac{\ln \langle T\rangle}{NL}, \qquad (1)$$

where Λ is the localisation length, NL is the length of the chain and T is transmittance [S2].

We started from the calculation of transmittance spectrum in 10 chain of disks. The localisation length is obtained by the averaging of transmittance over the 10 realisations as it was done in experiment. The obtained results are shown at Fig.S14. Since, if the wave is not localised, it completely passes from true the structure. Indeed, as it shown in Fig.S14 for the forbidden, e.i, waveguide modes frequencies one can see the maximums of the Λ, while for the frequencies above light line the values are small. Additionally, as the parameter $\sigma \geq 0.06$, the Λ values become less distinct. This indicates the emergence of hybrid states, characterized by localized features.

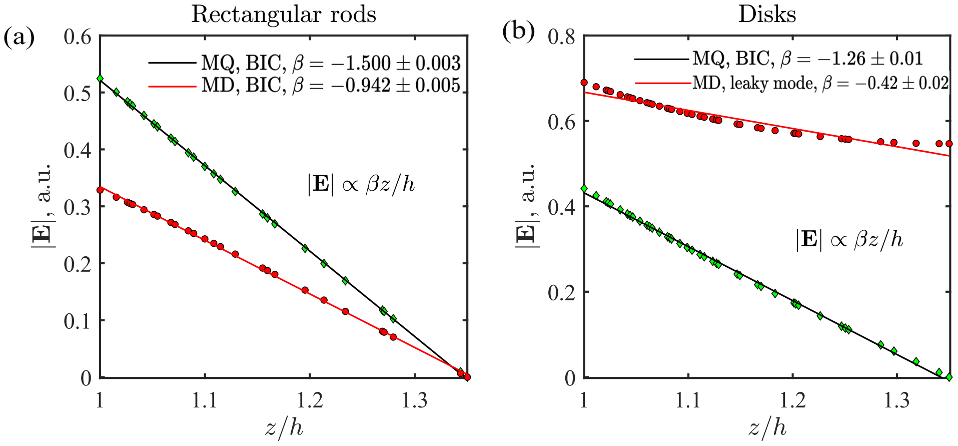

FIG. S12. Evolution of the field along the periodic direction z for quadrupole (MQ) and dipole (MD) modes.

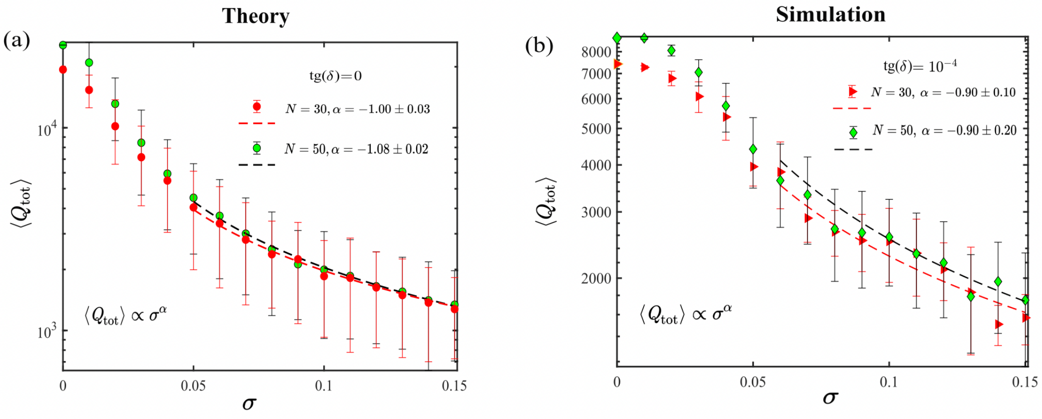

FIG. S13. (a) Theoretically obtained dependencies of the mean value of the total Q factor from the disorder parameter for a 300 different realizations; (b) Simulation results for a 100 realizations.

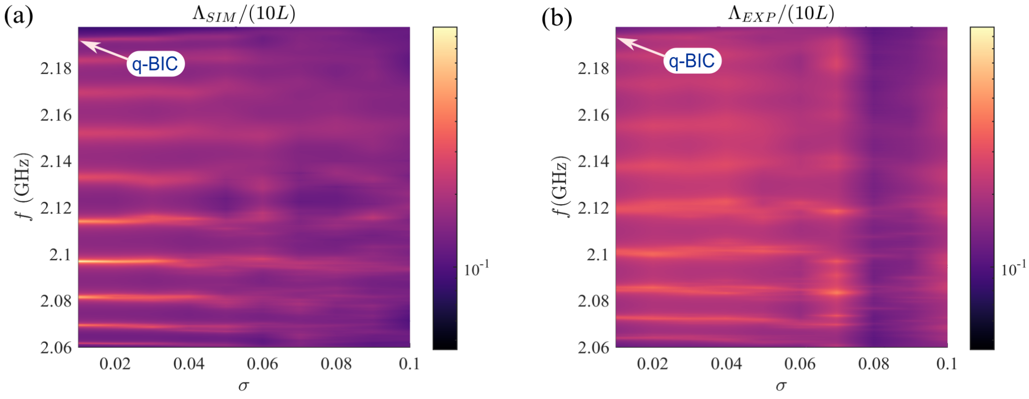

FIG. S14. Numerically calculated (a) and experimentally obtained (b) dependencies of normalized localization length Λ on the length of the chain NL from the disorder parameter σ in a 10-disk system.

X. EIGENFREQUENCY ANALYSIS

Although COMSOL Multiphysics is relatively easy to use, it allows solving a wide range of problems, including the eigenfrequency analysis. Despite the fact that the disks are three-dimensional objects, their symmetry and the

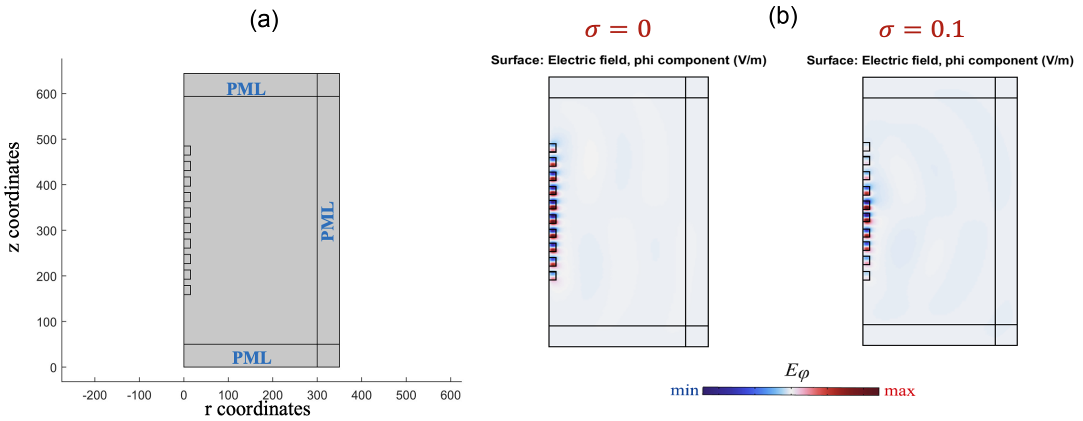

FIG. S15. (a) Geometry of the structure in COMSOL Multiphysics with the labeled position of PML. (b) Examples of eigenmodes for ordered and disordered chain.

longitudinal disorder enable us to reduce the problem to two dimensions. A method for simplifying 3D problems in the case of rotational symmetry can be found in Ref. [S3].

The geometry of the system that is used for eigenfrequency analysis in COMSOL Multiphysics is shown in Fig. S15(a). Owing to axial symmetry, the simulation was performed using a 2D cross-sectional model along the cylindrical axis. To simulate an open and nonreflecting infinite domain, a Perfectly Matched Layer (PML) was applied. The computational domain size was chosen carefully to avoid parasitic reflections from the PML boundary. As it is shown at Fig. S15(b), in the case of strong disorder, the mode begins to leak out. However, despite this leakage, due to the high dielectric permittivity, the field remains well localized within the chain region, which did not require additional optimization of the computational domain. Nevertheless, for small values of permittivity, the domain size was pre-optimized to ensure numerical stability and convergence of the solution (Fig. S5 (a)). Since we introduced randomly distributed disorder, the simulations were conveniently carried out using the COMSOL-MATLAB LiveLink interface, as described in detail in Section 3.1 of the main text.

S1. Maslova, E. E., Rybin, M. V., Bogdanov, A. A., & Sadrieva, Z. F. (2021). Bound states in the continuum in periodic structures with structural disorder. *Nanophotonics*, **10**(17), 4313–4321. De Gruyter.

S2. Poddubny, A. N., Rybin, M. V., Limonov, M. F., & Kivshar, Y. S. (2012). Fano interference governs wave transport in disordered systems. *Nature Communications*, **3**(1), 914.

S3. Gladyshev, Sergei, et al. Fast simulation of light scattering and harmonic generation in axially symmetric structures in COMSOL. *ACS Photonics 11.2* (2024): 404-418.
